# Epigenetic mechanisms linking hexavalent chromium exposure to pancreatic cancer risk: a systematic review

**DOI:** 10.1093/eep/dvag013

**Published:** 2026-04-10

**Authors:** Kai Hayashi, Yicong Huang, Kenneth R Muir, Artitaya Lophatananon

**Affiliations:** Division of Population Health, Health Services Research and Primary Care, School of Health Sciences, Faculty of Biology, Medicine and Health, The University of Manchester, Manchester M13 9PT, United Kingdom; Division of Population Health, Health Services Research and Primary Care, School of Health Sciences, Faculty of Biology, Medicine and Health, The University of Manchester, Manchester M13 9PT, United Kingdom; Division of Population Health, Health Services Research and Primary Care, School of Health Sciences, Faculty of Biology, Medicine and Health, The University of Manchester, Manchester M13 9PT, United Kingdom; Division of Population Health, Health Services Research and Primary Care, School of Health Sciences, Faculty of Biology, Medicine and Health, The University of Manchester, Manchester M13 9PT, United Kingdom

**Keywords:** epigenetics, chromium, pancreatic cancer, systematic review, genes

## Abstract

Pancreatic cancer remains one of the most lethal malignancies, largely due to late diagnosis and limited treatment options. Hexavalent chromium (Cr(VI)) is a well-established environmental and occupational carcinogen, and emerging epidemiological observations have raised questions about its potential involvement in cancers beyond the lung. This systematic review synthesizes current evidence on the epigenetic effects of Cr(VI) exposure, including DNA methylation, histone modifications, and microRNA dysregulation, and evaluates whether these mechanisms converge on pathways relevant to pancreatic carcinogenesis. A structured literature search of EMBASE and PubMed (including MEDLINE) identified 11 studies that met predefined inclusion criteria. Across these studies, Cr(VI) exposure was consistently associated with hypermethylation of tumour suppressor genes (including *MLH1* and *RAD51*), alterations in histone methylation (such as increased H3K9me2), and dysregulation of oncogenic microRNAs including miR-3940-5p. Collectively, these epigenetic alterations affect processes central to carcinogenesis, including DNA repair, genomic stability, inflammatory signalling, and cellular stress responses. Notably, several of the genes affected by Cr(VI) exposure such as *MLH1, RAD51, CD44*, and *Nupr1* are well-recognized contributors to pancreatic ductal adenocarcinoma (PDAC) development. Although none of the identified studies directly examined Cr(VI)-associated epigenetic changes in pancreatic tissue or pancreatic cell systems, the convergence of Cr(VI)-induced epigenetic alterations on molecular pathways central to PDAC biology highlights a set of biologically plausible and testable hypotheses linking chromium exposure to pancreatic cancer risk. Together, the available evidence suggests that Cr(VI) exposure could plausibly promote pancreatic carcinogenesis through epigenetic silencing of DNA repair pathways and activation of stress-response and stemness-associated signalling networks. By integrating findings across diverse experimental systems, this review identifies mechanistic intersections between chromium-induced epigenetic dysregulation and established drivers of PDAC. These findings highlight a clear opportunity for targeted investigation of Cr(VI)-associated epigenetic signatures in pancreatic-relevant experimental models and in chromium-exposed human populations. Such studies could determine whether environmentally induced epigenetic alterations contribute to pancreatic cancer susceptibility and may reveal previously under-recognized environmental drivers of pancreatic carcinogenesis.

## Introduction

Pancreatic cancer is projected to become one of the leading causes of cancer-related mortality, with a 5-year survival rate below 10% [1, 2]. While well-established risk factors include smoking, obesity, and type 2 diabetes [3], increasing attention is being paid to environmental and occupational exposures. Chromium, particularly in its hexavalent form (Cr(VI)), is a recognized carcinogen commonly encountered in industrial settings such as stainless steel production, leather tanning, and welding [4, 5]. Although chromium’s genotoxicity is well documented [6, 7], its epigenetic influence on cancer development remains underexplored.

Epigenetic mechanisms—such as DNA methylation, histone modifications, and non-coding RNA regulation—play a key role in modulating gene expression and are frequently disrupted in malignancies, including pancreatic ductal adenocarcinoma (PDAC) [8, 9]. This review aims to evaluate existing evidence on Cr(VI)-induced epigenetic alterations and explore their potential contribution to pancreatic cancer pathogenesis.

## Results

In total, 590 records were identified through comprehensive searches of the EMBASE and PubMed (including MEDLINE) databases. After removal of 378 duplicate records, a total of 212 unique articles remained for initial screening. These articles were assessed based on their titles and abstracts to determine their relevance to the research question.

Following this preliminary screening stage, 23 full-text articles were retrieved and reviewed in detail to assess eligibility against the inclusion and exclusion criteria. Of these, 11 studies met all criteria and were subsequently included in the final qualitative synthesis. A structured qualitative appraisal of methodological rigor across included studies is provided in Supplementary Tables S1 and S2. These studies comprised a mix of in vivo human observational studies and in vitro experiments using human-derived cell lines, all of which examined epigenetic effects attributable to hexavalent chromium (Cr(VI)) exposure. Of these, five were conducted in vivo using human tissue or blood samples from individuals with occupational chromium exposure, four were in vitro studies using immortalized human cell lines treated with Cr(VI) compounds, and two studies combined both in vivo and in vitro approaches to validate findings across biological systems. This diverse methodological mix provided a broad spectrum of evidence across different models of chromium exposure.

**Epigenetic alterations** induced by Cr(VI) exposure were consistently reported across all three major domains: DNA methylation, histone modification, and microRNA expression.

**DNA methylation** changes were among the most frequently observed. Several tumour suppressor and DNA repair genes—including *MLH1, HOGG1, RAD51*, and *CD44*—showed evidence of promoter hypermethylation in Cr(VI)-exposed cells and tissues. This hypermethylation was consistently associated with reduced mRNA expression, indicating transcriptional silencing. Given the functional roles of these genes in mismatch repair, oxidative damage response, and cell cycle regulation, their repression suggests a mechanism by which chromium exposure may contribute to genomic instability and tumorigenesis.

**Histone modifications** were also significantly affected. Multiple studies reported global increases in histone H3 lysine 9 dimethylation (H3K9me2) and lysine 27 trimethylation (H3K27me3), both of which are commonly associated with transcriptional repression. These chromatin-level changes occurred at gene loci involved in tumour suppression, suggesting that Cr(VI) may promote carcinogenesis by establishing a more repressive epigenetic landscape. Furthermore, increased acetylation at H3K9 and H3K14 observed at specific loci (e.g. *Nupr1*) indicates that Cr(VI) can also activate oncogenic pathways via histone acetylation.

**MicroRNA (miRNA) regulation** was less commonly studied but yielded notable findings. In particular, *miR-3940-5p* was found to be significantly downregulated in plasma from Cr(VI)-exposed individuals, which may affect tumour suppressor gene p53, leading to genomic instability and tumorigenesis. This miRNA was inversely correlated with the expression of a key DNA repair protein, *XRCC2*, implicating it in post-transcriptional enhancement of repair mechanisms. The dysregulation of miRNAs adds another layer of epigenetic disruption through which chromium may impair genomic integrity.

While none of the included studies investigated these epigenetic changes directly in pancreatic tissue or cell lines, several of the affected genes particularly *MLH1, RAD51, CD44*, and *Nupr1* are well recognized for their roles in PDAC pathogenesis. These findings suggest that the epigenetic mechanisms triggered by Cr(VI) in other tissues may involve pathways that are also central to pancreatic cancer biology, although direct evidence in pancreatic models is currently lacking, warranting further targeted investigation in pancreatic models.

## Discussion

This review synthesizes evidence that Cr(VI) exposure is associated with disruption of multiple epigenetic mechanisms, including DNA methylation, histone modification, and microRNA regulation, in human in vivo and human-derived in vitro systems [10–12]. Evidence linking chromium exposure directly to pancreatic cancer risk remains limited and largely epidemiological, and mechanistic epigenetic evidence in pancreatic tissue is currently lacking. The findings summarized here therefore provide insight into chromium-associated epigenetic alterations in human-relevant systems, rather than pancreas-specific mechanisms.

### Strength of evidence across epigenetic domains

DNA methylation was the most extensively investigated epigenetic domain. Multiple in vivo studies demonstrated hypermethylation of DNA repair and tumour suppressor genes, including *MLH1, HOGG1, RAD51*, and *CD44* following Cr(VI) exposure [11, 13–15]. These methylation changes were frequently accompanied by reduced mRNA expression, implying transcriptional silencing. MLH1 promoter hypermethylation was significantly associated with exposure duration in occupationally exposed individuals [15]. Given the established role of MLH1 in mismatch repair and genomic stability [13, 16], its epigenetic repression represents a plausible mechanism through which Cr(VI) exposure may contribute to carcinogenic processes.

Histone modification findings were more heterogeneous. Increased levels of H3K9me2 and H3K27me3 were reported following both acute and chronic Cr(VI) exposure [12, 17], marks typically associated with transcriptional repression at tumour suppressor loci. Divergent findings across exposure duration and cell type such as decreased H3K27me3 after short-term exposure [12,17] and increased levels after long-term exposure [18] suggest time-dependent chromatin dynamics. In addition, increased histone acetylation at loci such as Nupr1 indicates that Cr(VI) may also influence transcriptional activation pathways [10].

MicroRNA dysregulation was less extensively studied. Li *et al*. reported altered expression of miR-3940-5p in chromium-exposed individuals, associated with modulation of DNA repair-related proteins [18]]. Although limited in scope, this finding supports the hypothesis that post-transcriptional regulation may represent an additional epigenetic mechanism influenced by Cr(VI) [19].

### Critical appraisal of the evidence

Several limitations constrain interpretation of the current evidence base. First, none of the included studies examined Cr(VI)-induced epigenetic changes in pancreatic tissue or pancreatic cancer-specific models. Most evidence derives from lung tumour samples or bronchial epithelial cell lines such as BEAS-2B and 16HBE [10, 14], which may not reflect the epigenetic landscape of pancreatic tissue.

Second, substantial heterogeneity was observed in chromium compounds, exposure concentrations (0.25–20 μM), exposure duration (24 h to 40 weeks), and epigenetic endpoints [18, 20]. Several observational studies relied on occupational duration or job title as proxies for exposure, potentially introducing exposure misclassification [13, 20].

Third, functional validation was limited. Although epigenetic changes were frequently accompanied by altered gene expression, few studies demonstrated downstream phenotypic consequences or causal rescue experiments [11, 17]. Most studies also focused on a single epigenetic layer, limiting insight into cross-talk between DNA methylation, histone marks, and non-coding RNA regulation [21].

Finally, although animal models were excluded to preserve translational specificity, pancreas-specific rodent or transgenic models would provide important complementary mechanistic insight. Controlled in vivo systems could clarify whether chromium-associated epigenetic alterations observed in human-derived systems are recapitulated in pancreatic tissue and influence tumour initiation or progression.

### Implications for pancreatic cancer research

Although no pancreas-specific studies were identified, several chromium-associated epigenetic alterations affect genes and chromatin marks that are also implicated in PDAC biology. Epigenetic silencing of MLH1 and RAD51 may impair mismatch repair and double-strand break repair, processes central to genomic instability in PDAC [17, 22]. Suppression of HOGG1 may exacerbate oxidative DNA damage accumulation [12]. Similarly, altered expression of Nupr1 has been linked to stress adaptation and therapy resistance in PDAC [10, 23], while dysregulation of CD44 may influence tumour cell survival and invasion [14, 24]. Repressive histone marks such as H3K27me3, mediated by polycomb group proteins, are recognized contributors to pancreatic tumorigenesis [25].

These observations indicate mechanistic convergence between chromium-associated epigenetic alterations and pathways known to operate in pancreatic cancer. However, this convergence derives from non-pancreatic systems and should be interpreted cautiously.

It is important to distinguish between three levels of inference in this review. First, Cr(VI)-associated epigenetic alterations have been directly observed in human-exposed populations and human-derived cell systems. Second, many of the affected genes and chromatin modifications are known to play roles in general carcinogenic processes, including DNA repair impairment and chromatin repression. Third, extrapolation of these findings to pancreatic cancer remains hypothesis-generating, as no pancreas-specific experimental studies have yet been conducted. The relevance to pancreatic tumorigenesis should therefore be interpreted as biologically plausible but not experimentally established.

### Future directions

Future research should prioritize direct investigation of Cr(VI)-associated epigenetic alterations in pancreatic tissue or PDAC-relevant experimental models, including organoids and genetically engineered mouse systems. Prospective cohort studies incorporating high-resolution exposure assessment and biospecimen linkage may clarify epidemiological associations. Integration of multi-omics approaches could further elucidate interactions between DNA methylation, histone modification, and non-coding RNA regulation. In parallel, the development and application of genetic proxies for chromium exposure or biological response pathways could enable Mendelian randomization and related causal inference approaches in large population datasets. Such strategies would help determine whether chromium-related epigenetic changes lie on the causal pathway to pancreatic carcinogenesis. Together, these approaches are necessary to establish whether chromium-associated epigenetic signatures have translational relevance for risk stratification, early detection, or prevention in exposed populations.

## Materials and methods

### Study design

This review was conducted as a systematic analysis of the published literature, guided by the PRISMA 2020 (Preferred Reporting Items for Systematic Reviews and Meta-Analyses) statement [26]. The systematic review was registered with the International Prospective Register of Systematic Reviews (PROSPERO) under registration ID CRD420261294083. The objective was to identify, evaluate, and synthesize existing studies that investigated the epigenetic effects of Cr(VI) exposure, with particular interest in mechanisms relevant to carcinogenesis and potential implications for PDAC. By applying a structured review methodology, the study aimed to improve transparency and reproducibility, while critically appraising the scope and strength of the available evidence.

### Search strategy

A comprehensive search of the literature was conducted in February 2025 using two electronic databases: EMBASE and PubMed (including MEDLINE). These databases were selected for their wide coverage of biomedical and toxicological research. The search covered all available records up to the date of retrieval and was restricted to articles published in English.

A combination of controlled vocabulary terms and free-text keywords were used to optimize sensitivity. Boolean operators (AND, OR) and truncation symbols were applied to capture variations in terminology related to chromium exposure, epigenetic regulation, and cancer development.

Search Terms included (Chromium OR Chromate OR “Heavy Metals”) AND (“Epigenetics” OR “DNA Methylation” OR Histones OR “Histone Modification” OR “Gene Expression Regulation” OR “miRNA” OR “microRNA”) AND (“Neoplasm” OR “Cancer” OR Carcinogenesis OR “Pancreatic Cancer” OR “Cell Proliferation”). The full search strings were provided in the supplement material (S1). All search results were exported to a reference management system. Duplicate records were identified and removed before screening commenced.

### Eligibility criteria

Studies were selected based on predefined inclusion and exclusion criteria to ensure relevance and scientific rigor.

*Inclusion criteria:*

- Studies reporting epigenetic outcomes (e.g. DNA methylation changes, histone modifications, or alterations in microRNA expression) resulting from quantifiable Cr(VI) exposure
- Experimental or observational in vivo studies (human samples) or in vitro studies (human-derived cell lines)
- Peer-reviewed primary research articles
- Articles published in the English language

*Exclusion criteria:*

- Studies based on animal models, non-human cell lines, or mixed-metal exposure
- Use of Cr(III) without assessment of Cr(VI) effects
- Review articles, commentaries, editorials, or conference abstracts
- Studies without direct measurement of epigenetic outcomes
- Studies using misidentified cell lines (e.g. LO2)

### Study selection process

Two stages of screening were carried out:

1. Title and abstract screening for preliminary relevance
2. Full-text review to confirm eligibility based on inclusion criteria

**Figure 1:**
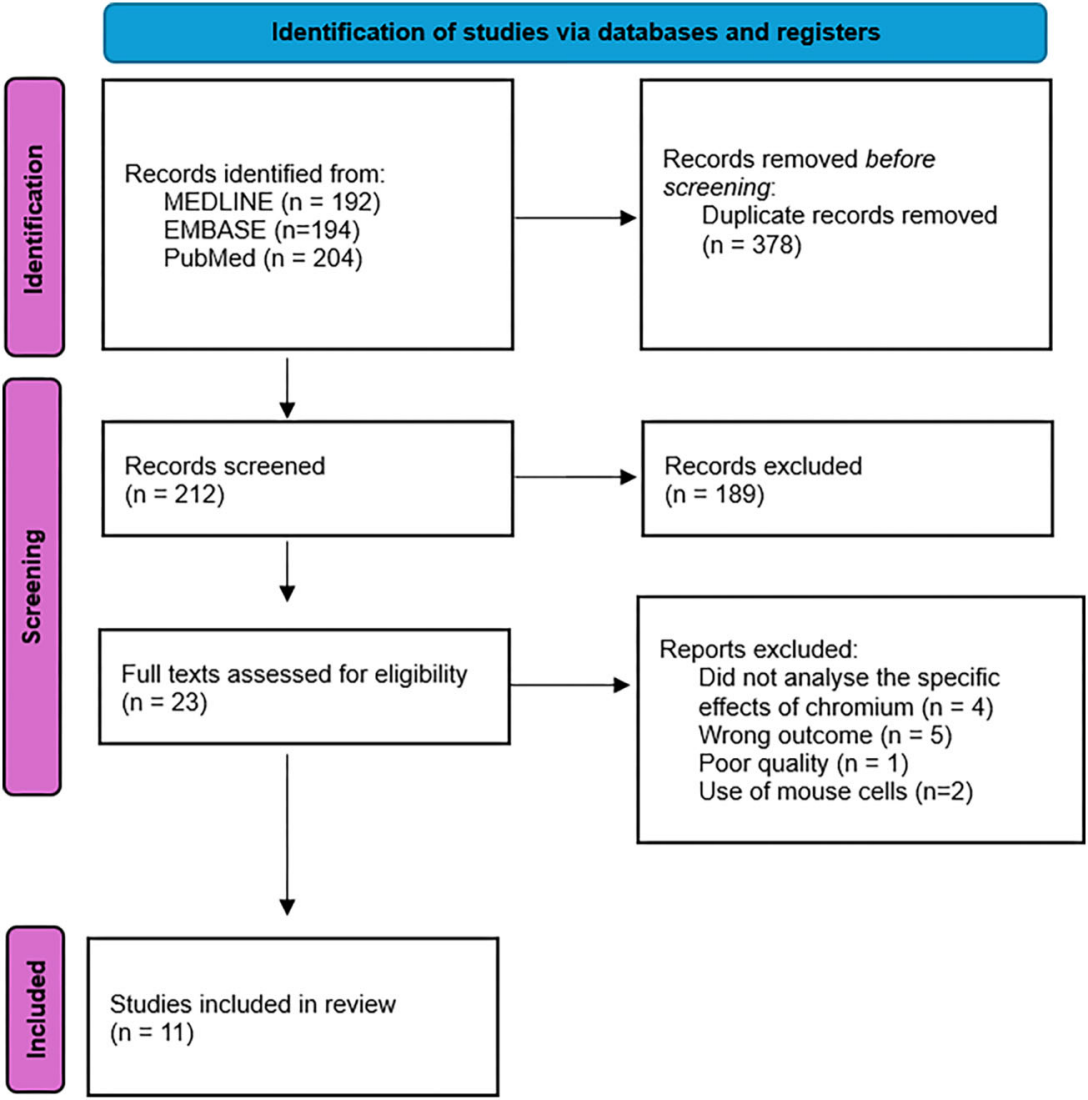
PRISMA flow diagram outlining the identification and selection of studies [26].

This process followed the PRISMA 2020 flow framework, and a flow diagram was developed to illustrate the identification, screening, eligibility assessment, and final selection of studies (Fig. 1).

### PRISMA flow diagram

#### Data extraction and synthesis

A structured data extraction template was used to collate the following information from each included study:

- Author and year of publication
- Study design and model system (in vitro or in vivo)
- Type and form of Cr(VI) exposure (e.g. K₂Cr₂O_7_, K₂CrO₄)
- Exposure dose and duration
- Targeted epigenetic outcome (e.g. gene-specific methylation, histone marks, miRNA profiling)
- Observed changes in gene or protein expression

The studies were categorized according to the primary epigenetic mechanism examined. They are DNA methylation, histone modifications, microRNA (miRNA) regulation.

Given the heterogeneity in experimental models, chromium formulations, and epigenetic endpoints, a narrative synthesis approach was used to summarize and interpret the findings rather than conducting meta-analysis.

#### Study quality and risk of bias assessment

Given the inclusion of both occupational observational studies and in vitro mechanistic experiments, no single validated risk-of-bias tool was appropriate across all study designs. Therefore, a structured qualitative appraisal framework was applied to enhance transparency in the assessment of methodological characteristics.

The appraisal was based on methodological features explicitly reported in the included studies and summarized in Tables 1 –3, including exposure measurement approach, type of epigenetic endpoint assessed, and evidence of downstream expression validation. Key study-level limitations identified in the *Discussion* section were also considered. Each study was systematically reviewed across these domains. The full structured appraisal is presented in the supplement material (S2).

**Table 1:** Summary of characteristics of in vivo studies.

Author (year)	Source of genetic material	Number of subjects/controls	Type of Cr(VI) exposure	Measure of Cr(VI) exposure	Epigenetic modification
Feng* et al*. [14]	Lung cancer	92/93	Occupational	Blood Cr, urinary Cr	DNA methylation
Hu* et al*. [11]	Lymphocytes isolated from venous blood	87/30	Occupational	Cr concentration of air in occupational environment, blood Cr	DNA methylation
Tsuboi* et al*. [15]	Lung cancer	32/31	Occupational	Duration of exposure	DNA methylation
Li* et al*. [18]	Plasma miRNA	87/30	Occupational	Blood Cr	miRNA
Ali *et al*. [13]	Lung cancer	36/25	Occupational	Duration of exposure	DNA methylation
Takahashi* et al*. [16]	Lung cancer	35/26	Occupational	Duration of exposure	DNA methylation
Kondo* et al*. [20]	Lung cancer	30/38	Occupational	Duration of exposure	DNA methylation

**Table 2:** Summary of characteristics of in vitro studies.

Author (year)	Cell type	Type of Cr(VI) exposure	Dosages of Cr(VI) (μM)	Duration of Cr(VI) exposure	Epigenetic modification
Hu* et al*. [11]	16HBE	K_2_Cr_2_O_7_	0.6, 1.2, 2.5, 5.0, 10.0, 20.0	24 h	DNA methylation
Feng* et al*. [14]	HMy2	K_2_Cr_2_O_7_	5, 10, 15	24 h	DNA methylation
			2.5	7 days	
Wang* et al*. [12]	BEAS-2B	K_2_Cr_2_O_7_	0.25	20 weeks	Histone methylation, histone acetylation
	16HBE			40 weeks	
Chen* et al*. [10]	BEAS-2B	K_2_CrO_4_	5, 10	24 h	DNA methylation, histone acetylation
			0.5	1 and 2 weeks	
Zhou* et al*. [27]	NHBE	K_2_CrO_4_	5, 10	24 h	Histone methylation
Sun* et al*. [17]	A549	K_2_CrO_4_	5, 10	1, 24, and 48 h	Histone methylation
	BEAS-2B		0.5, 1, 2, 5		

**Table 3:** Epigenetic changes induced by Cr(VI) and their associated effects.

Epigenetic changes	Gene/element	Epigenetic change induced by Cr (VI)	Effect
DNA methylation	MLH1 [13,15,16]	Increased in vivo with reduced mRNA expression	Suppressed DNA repair genes lead to genetic damage, mutation accumulation, and apoptosis avoidance, thereby increasing carcinogenesis
	MGMT, HOGG1, RAD51 [11]	Increased in vivo and in vitro with reduced mRNA expression	
	XRCC1, ERCC3 [11]	XRCC1 increased at CpG21 and decreased at CpG22/23, ERCC3 increased in vitro; mRNA expression reduced	
	CD44, SEMA4B [14]	Increased in vivo with reduced mRNA expression; no methylation change for CD44 in vitro with decreased mRNA expression	Facilitated carcinogenesis and promoted tumour growth through the PI3K/Akt pathway (SEMA48)
	p16 [20]	In vivo hypermethylation with reduced mRNA expression*	Silenced tumour suppressor gene, reduced protein expression, contributing to impaired cell cycle control
	APC [13]	In vivo hypermethylation; mRNA expression not assessed	Silenced tumour suppressor gene may lead to aberrant activation of the Wnt signal pathway and chromosomal instability
	Nupr1 [10]	Increased in vitro with increased mRNA expression	Elevated Nupr1 protein inhibited H4K16 acetylation and promoted anchorage-independent growth in BEAS-2B cells
Histone modification	H3K27me3 [12, 17]	Increased in BEAS-2B, 16HBE cells but decreases in A549 cancer cells	Condensed chromatin and MLH1 gene silencing results in cancer stem cell-like properties and cell transformation
	H3K9me2, H3K9me3 [12, 17]	H3K9me2 is globally increased in BEAS-2B, 16HBE, and A549, and at MLH1 in A549; H3K9me3 increased in BEAS-2B*, 16HBE	
	H3K4me2, H3K4me3 [27]	Globally increased in A549	Activate transcription
	H3K9ac, H3K14ac [10]	Increased in BEAS-2B, at Nupr1	Increased Nupr1 mRNA expression
miRNA regulation	miR-3940–5p [18]	Decreased in plasma; inversely correlated with increased XRCC2 protein expression.	Dysregulation may affect p53, contributing to genomic instability and carcinogenesis; mediated DNA repair through increased XRCC2 expression

## Conclusion

This review presents emerging evidence that Cr(VI) exposure induces significant epigenetic alterations including DNA hypermethylation, histone modifications, and microRNA dysregulation that may contribute to carcinogenesis through the repression of DNA repair and tumour suppressor genes, supporting a plausible role for Cr(VI) in carcinogenic processes.

However, the relevance of these findings to pancreatic cancer remains indirect and largely speculative. None of the available studies have examined Cr(VI)-induced epigenetic changes in pancreatic tissue or pancreatic cancer-specific models. Although several affected genes such as MLH1, RAD51, HOGG1, CD44, and Nupr1 are known to play key roles in pancreatic cancer biology, their involvement in chromium-associated pancreatic tumorigenesis has not been directly demonstrated through experimental or clinical validation in pancreatic models. Interpretation is further constrained by substantial heterogeneity in study design, limited functional validation, and a focus on single epigenetic layers in isolation.

Despite these limitations, the evidence reviewed here identifies credible epigenetic pathways through which environmental Cr(VI) exposure could contribute to pancreatic tumorigenesis. These mechanisms warrant further investigation using integrated, multi-omics approaches in pancreas-relevant models. Clarifying the epigenetic impact of chromium exposure in pancreatic tissue may lead to novel insights for early detection, prevention, and therapeutic targeting in at-risk populations.

## Supplementary Material

dvag013_Supplemental_File

## References

[1] Rahib L, Smith BD, Aizenberg R et al. Projecting cancer incidence and deaths to 2030: the unexpected burden of thyroid, liver, and pancreas cancers in the United States. Cancer Res. 2014;74:2913–21. 10.1158/0008-5472.CAN-14-0155 24840647

[2] Zottl J, Sebesta CG, Tomosel E et al. Unraveling the burden of pancreatic cancer in the 21st century: trends in incidence, mortality, survival, and key contributing factors. Cancers. 2025;17:1607. 10.3390/cancers17101607 40427106 PMC12110279

[3] Capasso M, Franceschi M, Rodriguez-Castro KI et al. Epidemiology and risk factors of pancreatic cancer. Acta Biomed. 2018;89:141–6. 10.23750/abm.v89i9-S.7923 30561407 PMC6502190

[4] IARC Monographs on the Evaluation of Carcinogenic Risks to Humans . Chromium, Nickel and Welding. 1990;vol 49:PMC7681426 2232124

[5] Shin DY, Lee SM, Jang Y et al. Adverse human health effects of chromium by exposure route: a comprehensive review based on toxicogenomic approach. Int J Mol Sci. 2023;24:3410. 10.3390/ijms24043410 36834821 PMC9963995

[6] DesMarais TL, Costa M. Mechanisms of chromium-induced toxicity. Curr Opin Toxicol. 2019;14:1–7. 10.1016/j.cotox.2019.05.003 31511838 PMC6737927

[7] O’Brien TJ, Ceryak S, Patierno SR. Complexities of chromium carcinogenesis: role of cellular response, repair and recovery mechanisms. Mutat Res. 2003;533:3–36. 10.1016/j.mrfmmm.2003.09.006 14643411

[8] Manic L, Wallace D, Onganer PU et al. Epigenetic mechanisms in metal carcinogenesis. Toxicol Rep. 2022;9:778–87. 10.1016/j.toxrep.2022.03.037 36561948 PMC9764177

[9] Liu P, Jacques J, Hwang CI. Epigenetic landscape of DNA methylation in pancreatic ductal adenocarcinoma. Epigenomes. 2024;8:41. 10.3390/epigenomes8040041 39584964 PMC11587027

[10] Chen D, Kluz T, Fang L et al. Hexavalent chromium (Cr(VI)) down-regulates acetylation of histone H4 at lysine 16 through induction of stressor protein NUPR1. PLoS One. 2016;11:e0157317. 10.1371/journal.pone.0157317 27285315 PMC4902237

[11] Hu G, Li P, Cui X et al. Cr(VI)-induced methylation and down-regulation of DNA repair genes and its association with markers of genetic damage in workers and 16HBE cells. Environ Pollut. 2018;238:833–43. 10.1016/j.envpol.2018.03.046 29627753

[12] Wang Z, Wu J, Humphries B et al. Upregulation of histone-lysine methyltransferases plays a causal role in hexavalent chromium-induced cancer stem cell-like property and cell transformation. Toxicol Appl Pharmacol. 2018;342:22–30. 10.1016/j.taap.2018.01.022 29391238 PMC5825290

[13] Ali AH, Kondo K, Namura T et al. Aberrant DNA methylation of some tumor suppressor genes in lung cancers from workers with chromate exposure. Mol Carcinog. 2011;50:89–99. 10.1002/mc.20697 21229606

[14] Feng L, Guo X, Li T et al. Novel DNA methylation biomarkers for hexavalent chromium exposure: an epigenome-wide analysis. Epigenomics. 2020;12:221–33. 10.2217/epi-2019-0216 31961222

[15] Tsuboi M, Kondo K, Soejima S et al. Chromate exposure induces DNA hypermethylation of the mismatch repair gene MLH1 in lung cancer. Mol Carcinog. 2020;59:24–31. 10.1002/mc.23125 31579968

[16] Takahashi Y, Kondo K, Hirose T et al. Microsatellite instability and protein expression of the DNA mismatch repair gene, hMLH1, of lung cancer in chromate-exposed workers. Mol Carcinog. 2005;42:150–58. 10.1002/mc.20073 15605365

[17] Sun H, Zhou X, Chen H et al. Modulation of histone methylation and MLH1 gene silencing by hexavalent chromium. Toxicol Appl Pharmacol. 2009;237:258–66. 10.1016/j.taap.2009.04.008 19376149 PMC2701251

[18] Li Y, Li P, Yu S et al. miR-3940-5p associated with genetic damage in workers exposed to hexavalent chromium. Toxicol Lett. 2014;229:319–26. 10.1016/j.toxlet.2014.06.033 24973494

[19] Pisano A, Sabalic A, Forte G et al. Relationship between heavy metals and miRNAs in pancreatic ductal adenocarcinoma. J Trace Elem Med Biol. 2025;92:127754. 10.1016/j.jtemb.2025.127754 41015025

[20] Kondo K, Takahashi Y, Hirose Y et al. The reduced expression and aberrant methylation of p16(INK4a) in chromate workers with lung cancer. Lung Cancer. 2006;53:295–302. 10.1016/j.lungcan.2006.05.022 16828922

[21] Zhao L, Islam R, Wang Y et al. Epigenetic regulation in chromium-, nickel- and cadmium-induced carcinogenesis. Cancers. 2022;14:5768. 10.3390/cancers14235768 36497250 PMC9737485

[22] Li M, Zhao ZW. Clinical implications of mismatched repair gene promoter methylation in pancreatic cancer. Med Oncol. 2012;29:970–76. 10.1007/s12032-011-9968-y 21660619

[23] Santofimia-Castano P, Xia Y, Peng L et al. Targeting the stress-induced protein NUPR1 to treat pancreatic adenocarcinoma. Cells. 2019;8:1453. 10.3390/cells8111453 31744261 PMC6912534

[24] Herrlich P, Morrison H, Sleeman J et al. CD44 acts both as a growth- and invasiveness-promoting molecule and as a tumor-suppressing cofactor. Ann NY Acad Sci. 2000;910:106–20.; discussion 118–20. 10.1111/j.1749-6632.2000.tb06704.x 10911909

[25] Neureiter D, Jager T, Ocker M et al. Epigenetics and pancreatic cancer: pathophysiology and novel treatment aspects. World J Gastroenterol. 2014;20:7830–48. 10.3748/wjg.v20.i24.7830 24976721 PMC4069312

[26] Page MJ, Moher D, Bossuyt PM et al. PRISMA 2020 explanation and elaboration: updated guidance and exemplars for reporting systematic reviews. BMJ. 2021;372:n160. 10.1136/bmj.n160 33781993 PMC8005925

[27] Zhou X, Li Q, Arita A et al. Effects of nickel, chromate, and arsenite on histone 3 lysine methylation. Toxicol Appl Pharmacol. 2009;236:78–84. 10.1016/j.taap.2009.01.009 19371620 PMC2684878

