# Supplementary material for: Epigenetic mechanisms linking hexavalent chromium exposure to pancreatic cancer risk: a systematic review
Source: Environ Epigenet. 2026 Apr 10;12(1):dvag013. doi: 10.1093/eep/dvag013 (PMC13109720; doi:10.1093/eep/dvag013)
Supplement: dvag013_Supplemental_File [file dvag013_supplemental_file.docx]

**Supplement 1: Search Strings**

A comprehensive search of the literature was conducted in February 2025 using two electronic databases: EMBASE and PubMed (including MEDLINE). These databases were selected for their wide coverage of biomedical and toxicological research. The search covered all available records up to the date of retrieval and was restricted to articles published in English.

A combination of controlled vocabulary terms and free-text keywords were used to optimise sensitivity. Boolean operators (AND, OR) and truncation symbols were applied to capture variations in terminology related to chromium exposure, epigenetic regulation, and cancer development.

The following key words were used in EMBASE to extract all relevant literature (1 AND 2 AND 3):

1. Chromium OR Chromate OR Metals OR Heavy Metals
2. Epigenetic OR Epigenomic OR Epigenome OR Gene Expression Regulation OR Gene Amplification OR Gene Silencing OR DNA Methylation OR Histones OR Chromatin
3. Neoplasms OR Cancer OR Carcino* OR Cell Division OR Cell Proliferation

Search Terms in PubMed included (Chromium OR Chromate OR "Heavy Metals") AND

("Epigenetics" OR "DNA Methylation" OR Histones OR "Histone Modification" OR "Gene Expression Regulation" OR "miRNA" OR "microRNA") AND ("Neoplasm" OR "Cancer" OR Carcinogenesis OR "Pancreatic Cancer" OR "Cell Proliferation").

**Supplementary Methods: Structured Qualitative Appraisal Framework**

A structured qualitative appraisal was conducted to enhance transparency in the assessment of methodological rigor across included studies. Domains were defined prior to structured evaluation and were selected based on methodological features consistently reported in the included literature and relevant to environmental epigenetic research.

For observational studies, appraisal focused on exposure measurement approach, type of epigenetic endpoint assessed, and evidence of downstream functional validation. The quality control criteria of observational studies are as follows:

1. Exposure measurement approach

High quality

- Individual-level Cr (VI) exposure quantified using validated biomarkers (e.g. blood or urinary chromium).

Low quality

- Single time-point exposure assessment for a biologically cumulative exposure.

2. Type of epigenetic endpoint assessed

High quality

- Genome-wide profiling or multi-layer epigenetic assessment.

Low quality

- Candidate-gene methylation analysis without justification for gene selection or single-layer analysis only.

3. Evidence of downstream functional validation

High quality

- Demonstration that differential methylation is accompanied by concordant changes in mRNA or protein expression in the same individuals; Correlation analyses linking epigenetic markers to gene expression levels.

Low quality

- No expression assessment or unlinked datasets.

For in vitro studies, appraisal focused on exposure description (dose and duration), epigenetic endpoint type, and evidence of downstream functional validation. The quality control criteria of in vitro studies are as follows:

1. Exposure description (dose and duration)

High quality

- Multiple Cr (VI) concentrations with clearly defined exposure durations.

Low quality

- Exposure duration not reported or inconsistently described.

2. Epigenetic endpoint type

High quality

- Multi-layer epigenetic assays; Use of validated, quantitative assays (e.g. bisulfite sequencing, ChIP-qPCR/ChIP-seq).

Low quality

- Indirect or non-specific epigenetic measures.

3. Evidence of downstream functional validation

High quality

- Demonstration that differential methylation is accompanied by concordant changes in mRNA or protein expression in the same individuals; Correlation analyses linking epigenetic markers to gene expression levels.

Low quality

- Reporting epigenetic alterations without testing their effect on gene expression or cellular function

In addition to the predefined high- and low-quality descriptors for each methodological domain, an intermediate moderate category was applied when study characteristics fell between these criteria. This occurred when studies demonstrated partial adherence to high-quality features but retained notable methodological limitations (e.g., biomarker-based exposure measurement but candidate-gene epigenetic analysis, or evidence of gene expression changes without protein validation).

In an overall methodological concern was determined by qualitative integration of the three appraisal domains (exposure measurement, epigenetic endpoint, and functional validation). Studies were classified as:

- Moderate concern: at least two domains rated moderate or high with no domain rated low.
- Low–moderate concern: one domain rated moderate and two domains rated low, or studies showing predominantly lower-quality features but with some methodological strengths.
- Moderate–high concern: two or more domains rated high but with remaining methodological limitations (e.g., lack of protein validation or single dose exposure).

This classification reflects the overall balance of methodological strengths and limitations rather than a numerical scoring system.

Assessment was based exclusively on methodological characteristics explicitly reported in the included studies and summarised in the main manuscript (Tables 1–3). No additional assumptions were made beyond reported study characteristics.

Supplementary Table S1: Structured qualitative appraisal of included in vivo (observational) studies based on reported methodological characteristics.

| **Study** | **Exposure Measurement (Reported in Table 1)** | **Epigenetic Endpoint** | **Functional Validation Reported** | **Key Limitation Noted in Manuscript** | **Overall Risk of Bias (combined domain assessment)** |
| --- | --- | --- | --- | --- | --- |
| **Hu et al. (2018)** | Moderate: Air Cr + Blood Cr | Moderate: Candidate-gene analysis with justification (MGMT, HOGG1, XRCC1, ERCC3, and RAD51) | High: Reduced mRNA expression reported and measured chromosomal damage markers | Cross-sectional design; small Sample size; Candidate-gene only | Moderate |
| **Feng et al. (2020)** | Moderate:  Blood Cr, Urinary Cr; Cross-sectional design | High: DNA methylation (EWAS) + targeted bisulfite sequencing | Moderate: Reduced mRNA expression reported | Cross-sectional design; Small sample size; No protein expression measurement | Moderate |
| **Tsuboi et al. (2020)** | Low: Duration of exposure | Moderate: Candidate-gene analysis with justification (MLH1) | Moderate: Reduced MLH1 protein levels reported | Cross-sectional design; Small sample size; Exposure proxy measurement | Moderate |
| **Ali et al. (2011)** | Low: Duration of exposure | Low: Candidate-gene analysis without justification (APC, MGMT, hMLH1) | Moderate: mRNA expression was partially assessed | Cross-sectional design; Small sample size; Exposure proxy measurement; No protein expression measurement | Low-Moderate |
| **Takahashi et al. (2005)** | Low: Duration of exposure | Low: Candidate-gene analysis without justification (hMLH1 promoter) | Moderate: mRNA expression was not measured; Loss of hMLH1 protein expression reported | Cross-sectional design; Small sample size; Exposure proxy measurement; Limited confounder reporting | Low-Moderate |
| **Kondo et al. (2006)** | Low: Duration of exposure | Moderate: Candidate-gene analysis with justification (p16) | Moderate: Reduced p16 protein reported | Exposure proxy measurement; Small sample size; single gene focus | Moderate |
| **Li et al. (2014)** | Moderate: Blood Cr | Moderate:  miRNA microarray + qRT-PCR (miR‑3940‑5p) | Moderate: Protein correlation (XRCC2) | Cross-sectional design; Small sample size; Single miRNA focus | Moderate |

Supplementary Table S2: Structured qualitative appraisal of included in vitro studies based on reported methodological characteristics.

| **Study** | **Exposure Description (Dose & Duration Reported)** | **Epigenetic Endpoint** | **Downstream Validation Reported** | **Key Limitation Noted in Manuscript** | **Overall Risk of Bias (combined domain assessment)** |
| --- | --- | --- | --- | --- | --- |
| **Hu et al. (2018)** | Moderate: Controlled dosing  (0.6–20μM, 24 hours | Moderate: Candidate-gene analysis with justification (MGMT, HOGG1, XRCC1, ERCC3, and RAD51) | Moderate: Reduced mRNA expression reported | Short exposure duration; Candidate-gene only | Moderate |
| **Feng et al. (2020)** | High:  Controlled dosing  (5–15μM, 24 hours; 2.5μM 7 days) | High:  DNA methylation (EWAS) + targeted bisulfite sequencing | Moderate: Reduced mRNA expression reported | No protein expression measurement | Moderate-High |
| **Wang et al. (2018)** | Moderate: Only one chronic dose (0.25 μM, 20–40 weeks) | High: Histone modification H3K9me2, H3K27me3, H3K4me2, H3K36me2, H3K79me2, H3 acetylation) + Histone methyltransferases (G9a, GLP, SUV39H1, EZH2) | Knockdown experiments showed CSC-like property and cell transformation, and DNA damage | Only one chronic dose | Moderate-High |
| **Chen et al. (2016)** | High: Controlled dosing (5–10 μM 24 hours; 0.5 μM 1-2 weeks) | High: Histone modification (H3K9ac, H3K14ac) + DNA methylation (Nupr1) | Moderate: Increased Nupr1 mRNA reported | Single cell line model | Moderate-High |
| **Zhou et al. (2009)** | Moderate: Controlled dosing (5–10 μM 24 hours) | Moderate: Histone modification (H3K4me2, H3K4me3) | Low: No gene expression or functional assays | Short exposure duration; No functional validation | Moderate |
| **Sun et al. (2009)** | Moderate: Controlled dosing (5–10 μM; 1–48 h) | High: Histone methylation (H3K9me2, H3K9me3, H3K27me3) + Gene‑specific promoter analysis (MLH1) | Moderate: Reduced MLH1 mRNA reported | Short exposure duration | Moderate |
